# Workplace wellbeing among health care workers providing HIV services in primary care in Johannesburg: a mixed methods study

**DOI:** 10.3389/fpubh.2023.1220301

**Published:** 2023-10-20

**Authors:** Ndinda Makina-Zimalirana, Melanie Bisnauth, Nosipho Shangase, Natasha Davies, Anele Jiyane, Fezile Buthelezi, Kate Rees

**Affiliations:** ^1^Anova Health Institute, Johannesburg, South Africa; ^2^Department of Community Health, School of Public Health, Faculty of Health Sciences, University of the Witwatersrand, Johannesburg, South Africa

**Keywords:** burnout, healthcare, primary health care, wellbeing, healthcare worker (HCW)

## Abstract

**Background:**

Burnout among Health Care workers (HCWs) impacts on provider-patient relations and quality of care. Anova Health Institute (Anova) is a large South African non-profit organization and PEPFAR/USAID implementing partner. We conducted a study among HCWs providing HIV-related services in primary care settings in Johannesburg, South Africa, to examine levels of burnout, understand factors affecting workplace wellbeing, and explore strategies to prevent burnout.

**Methods:**

We used a sequential mixed-method approach. Data were collected between February and April 2022. The first phase consisted of a survey using the Maslach Burnout Inventory Human Services Survey (MBI-HSS) to measure levels of burnout. We then interviewed a subset of survey participants to understand the experiences that may affect wellbeing. We used descriptive statistics to quantify burnout rates for each MBI dimension (emotional exhaustion, personal accomplishment, and depersonalization). Qualitative data analysis was guided by the Job Demands-Resources Framework that explores the interactions between demands and resources in the workplace.

**Results:**

Survey findings (*n* = 194) revealed that although depersonalization rates were low at 6%, 21% of participants had high emotional exhaustion and 24% reported low professional accomplishment. Less than half (41%, *n* = 80) had scores in the high category for any one of the three MBI-HSS dimensions. The MBI-HSS dimensions differed significantly by type of work and job title. Roving positions (HCW working in more than one health facility) were more likely to experience higher emotional exhaustion and lower professional accomplishment. Qualitative findings (*n* = 25) indicate that a number of job demands, including high workload, inadequate mental health support, and challenging relationships with stakeholders, had a negative effect on HCWs’ wellbeing. However, finding meaningfulness in their work, working as a team, and practicing autonomy were experienced as resources that reduced the negative effect of these demands.

**Conclusion:**

While measured burnout syndrome rates were low, various experiences negatively impacted at least one in five HCW. We identified key resources that provided buffering against workplace stressors. We recommend that as well as addressing key drivers of burnout, access to these resources should be fostered, for example by strengthening interventions that offer recognition, and promoting team interactions through social activities and support groups.

## Introduction

Burnout has become an important challenge for public health and is recognized as a prevalent occupational hazard among healthcare workers (HCWs) globally (1). Burnout is characterized by emotional exhaustion, depersonalization, and reduced personal accomplishment (2). Research in high-income countries has revealed that as many as 50% of primary care providers report high levels of emotional exhaustion, depersonalization, and diminished personal accomplishment (3, 4). Although limited information on burnout from low- and middle-income countries (LMICs) has been reported (5), research reveals burnout rates of 50.1% among primary HCWs in China (6) and 51% in Brazil (7). Within Sub-Saharan Africa, burnout prevalence varies from 40 to 80% among HCWs, and had been attributed to adverse work conditions, high job demands, and low job satisfaction (8).

The consequences of burnout pose significant concerns for HCWs, clients, and the health system (9–12). HCWs’ motivation, retention, and compassionate care can be impacted, influencing patient interactions and the provision of quality healthcare (11, 12).

The COVID-19 pandemic exacerbated the mental health burden on HCWs (13–16), impacting their psychological wellbeing (14, 17). In South Africa, scarce resources, combined with the added strain of the pandemic on an already vulnerable healthcare system, seem to have significantly heightened the stress encountered by HCWs during this period (18). Furthermore, intensified health needs for patients with tuberculosis (TB) and HIV, and strict lockdowns have further amplified socio-economic challenges, adding pressure to an already strained healthcare system, particularly affecting the public sector (19).

HCWs employed by non-profit organisations (NPO) play a crucial role (20, 21), especially in under-resourced settings where there is a shortage of HCWs employed by the Department of Health. While South Africa has relatively higher HCW densities than other SSA countries, the demand remains unmet, particularly within the Johannesburg Metropolitan area, which houses 10% of the country’s population (22). The country’s high burden of people living with HIV (PLHIV) places added strain on public primary healthcare services, necessitating the involvement of NPO-employed HCWs (23). Promoting the wellbeing of NPO-employed HCWs presents unique challenges due to the unpredictable nature of funding and competing priorities (24).

However, there is a research gap on burnout in primary health care settings in LMICs, particularly in the HIV/TB-funded NPO sector, and there is limited mixed methods research on HCWs’ burnout (15, 25). To bridge this gap, the Anova Health Institute, a South African NPO, conducted a mixed methods study aiming to measure burnout, understand factors affecting workplace wellbeing, and explore strategies to mitigate burnout among HCWs employed by Anova, thereby contributing to a better understanding of HCWs’ wellbeing in the context of NPOs and primary care settings.

## Materials and methods

The study was conducted amongst Anova employed HCWs providing or supporting services in Johannesburg Metropolitan area in 127 primary healthcare clinics. According to data from the District Health Information System spanning April 2021 to March 2022, clinics had an average quarterly PHC headcount ranging from 1,600 to 20,300 people, inclusive of those on antiretroviral therapy and utilizing HIV services.

### Study design

This study used sequential mixed methods design, combining quantitative and qualitative approaches. The quantitative approach allowed the authors to measure levels of burnout using a validated tool. The qualitative data from interviews added depth to the initial quantitative findings from questionnaires (26), by explaining what is causing burnout, what is protecting people from burnout, and how their experience of the workplace either leads to burnout or does not. This integration harnesses the complementary nature of both approaches, addressing the limitations and enhancing the strengths of each (27). Qualitative methodologies strengthen quantitative findings by allowing researchers to explore associations identified in quantitative data (26). The first phase consisted of a survey to measure burnout. A survey was chosen as it allows large populations to be assessed with relative ease (28). A validated survey instrument, the Maslach Burnout Inventory Human Services Survey (MBI-HSS) (2) was used. The MBI-HSS was previously validated in South Africa among emergency medical service (29) and in a multi country study among nurses that included countries from Africa (30). MBI-HSS consists of three component scales: emotional exhaustion (9 items), depersonalization (5 items) and personal accomplishment (8 items), each measuring a dimension of burnout. All MBI-HSS items were scored using 7 level frequency ratings from “never” to “daily” (2, 31).

The second phase was a qualitative approach. We conducted in-depth interviews with a sub-set of survey participants. This phase sought to explain key survey findings and explore factors affecting workplace wellbeing, to inform activities for preventing burnout in the future.

### Study population and sampling

All HCWs working in Anova Johannesburg program teams in the organization were eligible, including those in primary contact with patients such as clinicians, lay counselors, and administrative staff, and those whose tasks do not involve primary contact with patients, such as monitoring and evaluation and support staff. Approximately 973 staff members including facility based and support staff in Johannesburg were invited to participate in the voluntary electronic survey.

Following analysis of the MBI-HSS survey data, a sub-set of respondents were selected for a follow up in-depth interview, from those who agreed to be contacted during the survey. Interviews were conducted purposively to ensure individuals from different facilities as well as clinical (e.g., nurse) and non-clinical (e.g., data capturer) HCWs were included. Participants were interviewed until “theoretical saturation” was achieved, and no new themes or issues appeared in the data collected (32).

### Data collection

#### Quantitative: surveys

A survey was distributed to 973 Anova HCWs between February and March,2022. Table 1 below shows the characteristics of HCWs who submitted survey responses.

**Table 1:** Health care workers characteristics.

	Population (all Anova Johannesburg employees)		Survey respondents		Qualitative interviews participants	
Characteristics	*N*	%	*N*	%	*N*	%
Gender						
Female	711	73%	152	78%	18	72%
Male	262	27%	40	21%	7	18%
Prefer not to say			2	1		
Job title						
Counsellor (HIV testing and adherence)	330	34%	79	41%	11	44%
Data (Data Capturer and M&E Officer)	195	20%	38	20%	3	12%
Professional or enrolled nurse	128	13%	36	19%	4	16%
Decanting	57	6%	11	6%	3	12%
Other	263	27%	30	16%	4	16%

An invitation to complete the survey and follow-up reminder (within a 4-week period) was sent out through work email addresses. Team managers were also asked to communicate to teams verbally about the survey in case the emails were not accessed. Survey data was collected and managed using a secure, web-based Research Electronic Data Capture (REDCap) (33), program. A paper-based option was made available. Participation in this study was voluntary and confidential. All the participants were > 18 years and were able to provide informed consent and were assured that the information would remain confidential (that no one other than the research team would know their identity) and that only anonymous quotes would be included in any resultant reports. HCWs were invited to participate in the study, giving participants an opportunity to decline without feeling pressured and assured no repercussions as a consequence of their response. Additionally, participants were given information on where they can seek help regarding their mental wellbeing.

Both electronic and paper-based surveys asked participants if they would like to participate in a follow-up interview. If they agreed to take part and provided contact details, and purposively selected by the study team to be interviewed, an appointment would be set to conduct the interview.

#### Qualitative: semi-structured interviews

We used existing literature on factors in the work environment known to affect burnout (34, 35) to develop the interview guide (Supplementary File S1). The guide discussed how participants experience and perceive burnout-related factors, e.g., autonomy, team dynamics, workload, support, feedback and their coping mechanisms. The interview guide was piloted, and only minor changes were made, focusing on improving the order of questions. The interviews were between 30 and 90 min. Interviews were conducted in person in isiZulu, SeSotho, TshiVenda, and English, audio recorded, translated to English, and transcribed. Transcripts were not returned to participants for comment.

### Data management and analysis

#### Surveys

Survey data was analyzed using Stata 14 (StataCorp. 2015. Stata Statistical Software: Release 14. College Station, TX: StataCorp LP, TX, USA). Descriptive statistics were calculated, and multivariable logistic regression analysis was used to investigate the relationship between high scores for each dimension and possible explanatory factors. Explanatory factors were gender, place of work [Community Health Centre (CHC), Primary Health Care (PHC), community work, hospital, or office], roving (HCW supporting more than one health facility), clinical (e.g., professional nurse, enrolled nurse) vs. non-clinical staff, and level of job role [1 = frontline staff; 2 = frontline staff managers (e.g., sub-district level manager); 3 = management level above sub-district level 2 (e.g., district team level manager); 4 = senior program management].

#### Burnout measures

The dimensions were categorized into 3 levels (low, moderate or high) based on the reference ranges provided with the MBI-HSS: emotional exhaustion scores were categorized as low (0–16), moderate (17–26) and high (≥27), professional accomplishment scores were categorized as low (0–31), moderate (32–38) and high (≥39), and depersonalization scores were categorized as low (0–6), moderate (7–12) and high (≥13) (2). Low scores are preferred for emotional exhaustion and depersonalization, while high scores are ideal for professional accomplishment.

### Semi-structured interviews

#### Theoretical framework

We used a framework that tackles the interactions between demands and resources in the workplace context: the Job Demands-Resources (JD-R) model (35, 36). The JD-R model is one of the most applied models of burnout and work stress, specifically applicable when seeking to better understand the dynamics of wellbeing and motivational engagement processes in a demanding work environment (37–39). According to the model, an accumulation of demands (e.g., time pressure, unfavorable environmental conditions, interpersonal conflict, abusive supervision) places a strain on mental health, leading to negative outcomes such as exhaustion or burnout (34). On the other hand, resources (e.g., autonomy, support, feedback) have a positive impact and result in higher engagement and better performance at work through a motivational process (37). Demands and resources also interact with one another, meaning that available resources can soften the negative impact of demands (*ibid.*). The model assumes that high demands require workers to make more effort, resulting in diverse coping behaviors (34).

### Analysis

Audio recordings were transcribed, with simultaneous translation. Transcripts were imported into NVivo 12 (NVivo qualitative data analysis software; QSR International Pty Ltd. Version 12, 2018). This software was used to organize and classify the data. Data were analyzed inductively using thematic analysis (40). The coding took place in two steps: the first team generated initial codes, and categories in an exploratory manner, while the second team independently reviewed and validated these codes and categories. During step 1, NMZ and KR, conducted open coding, during which the authors examined the text line by line and assigned codes to different parts of the data by marking and comparing meaning units and sorted them into codes. These codes were preliminary, and the coding process was iterative. NMZ conducted the second phase which involved categorization, where the codes were grouped into sub-categories and these sub-categories were then organized into broader categories. Each sub-category consisted of codes that were related in terms of topic or concept. Throughout this process, categories and codes were continually reviewed, leading to adjustments in naming, combining, and re-categorizing. Through this reanalysis, patterns and themes in the data were developed. To enhance the reliability of the findings, AJ and FB reviewed the coding process to ensure consensus on the identified codes, categories, and themes. Finally, themes were mapped to the JD-R model components: job demands. Job resources, and coping strategies.

## Results

### Survey results

A total of 194 HCWs participated in the survey, representing a response rate of 20% (194/973) and a good representation of our population was achieved (Table 1). There were more female than male HCW in the organization (73% vs. 27%), and in the participants who completed the survey (78% vs. 22%). Similarly, within the job category, the proportion within each category mirrored the organization distribution. Among the participants, most were female (78%, *n* = 152), and were non-roving, i.e., based at a single facility (79%) (Table 2). Highly represented job titles were counselors [HIV testing and (antiretroviral therapy) ART adherence counselors] (41%), data capturers (20%) and professional or enrolled nurses (19%).

**Table 2:** Characteristics of health care workers who scored EE = high, D = High, PA = Low.

	All participants		EE = high or D = high or PA = Low			EE = high			PA = Low			D = high		
Characteristics	*n*	%	*n*	%	*p*	*N*	%	*p*	*n*	%	*p*	*n*	%	*p*
Total	192		80	41		41	21		47	24		12	6	
Gender														
Female	152	78	66	43	0.28	33	22	0.67	38	25	0.68	9	6	0.72
Male	40	21	13	33		7	18		8	20		3	8	
Prefer not to say	2	1	1	50		1	50		1	50				
Roving														
No	153	79	56	37	0.02	27	18	0.05	33	11	0.10	10	7	0.99
Yes	40	20	23	58		13	33		14	35		2	5	
Job title														
Counselors	79	41	21	27	0.01	10	13	0.01	9	11	0.01	6	8	0.01
Data team	38	20	26	68		12	32		22	58		1	3	
Professional/enrolled nurse	36	19	10	28		5	14		5	14		3	8	
Decanting Mentor/Coordinator	11	6	6	55		1	9		5	45			0	
Other	30	16	16	53		12	40		6	20		2	7	

EE, emotional exhaustion; PA, professional accomplishment; D, depersonalization. *p*-values: Fishers exact test.

**Figure 1:**
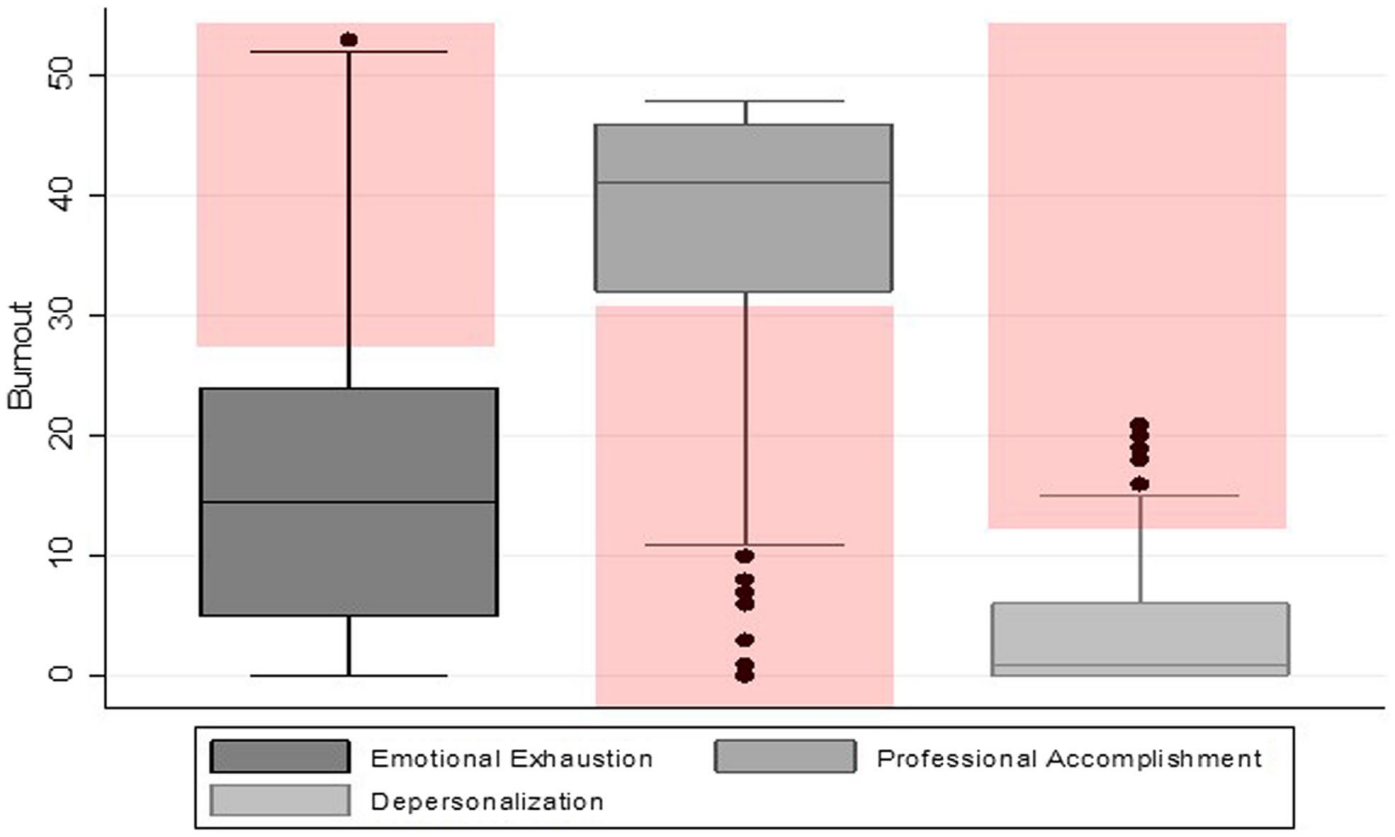
Distribution of burnout dimension scores. Red shading indicates high risk in each dimension.

MBI-HSS survey analysis revealed a low overall burnout rate of 0.5% across the 194 respondents but 40% of HCWs reported moderate/high levels of emotional exhaustion (Supplementary File S2). The scores for the three dimensions are represented in Figure 1. The median score for emotional exhaustion was 14.5 (low) with a range of 0–53.0. For professional accomplishment, participants had a median of 41.0 (high), which ranged from 0 to 48.0 and for depersonalization was 1 (low) with a range of 0–21.0.

In total 41% of the participants were categorized as either high emotional exhaustion or reduced personal accomplishment or high depersonalization. The characteristics of those who scored negatively for each MBI-HSS dimension among the participants is presented in Table 2. The comparison among different dimensions of burnout indicated that the proportion of reduced personal accomplishment (24% = 47/194) and emotional exhaustion (21% = 41/194) is relatively higher than depersonalization (6% = 12/194) among the participants.

The results revealed that the emotional exhaustion, professional accomplishment, and depersonalization of participants were not significantly different by gender. A significantly higher proportion of roving positions (HCW supporting more than one health facility) than non-roving reported high emotional exhaustion (67.5% vs. 32.5%, *p* = 0.02) and lower professional accomplishment (70.2% vs. 29.8%, *p* = 0.01). Depersonalization scores of participants were not significantly different by their roving status. The results revealed that the feeling of reduced personal accomplishment and depersonalization significantly differed by job titles. In depersonalization, nurses and counselors had higher scores than other job categories (*p* = 0.01%), while counselors and data capturers had reduced personal accomplishment (*p* = 0.01%).

### Qualitative results

Twenty-five HCWs were interviewed. Table 1 describes the interviewees’ characteristics.

Overall burnout syndrome levels among the participants were low. Given this, we centered our qualitative analysis on understanding the interaction between job demands and workplace wellbeing, to identify potential methods for promoting wellbeing in healthcare settings. Additionally, given the relatively higher likelihood of emotional exhaustion and low professional accomplishment among roving staff, our qualitative analysis looked for a possible explanation. However, we did not find any substantial differences in responses between roving HCWs and non-roving.

We developed five themes that contributed (positively or negatively) to work wellbeing: work conditions, management practices, work relationships, meaningfulness of work, and personal factors. Table 3 presents the themes and subthemes representing key job demands and resources.

**Table 3:** Summary of themes identified in the analysis, organized into job demands and resources.

Themes and subthemes	Job demand – description	Job resource -description
**Theme – working conditions**		
Mental health support	Lack of activities to support employees’ mental wellbeing	
Workload	High workload has a negative impact on HCW wellbeing	
Work life balance	Inability to balance and separate work and private life	
Emotional effect of interacting with patients	Coping with distressing clients especially those connected to children, violent crimes, and sexual violence	
Nature of funding	Unpredictable funding in the sector affecting job security	
**Theme: work relationships**		
Colleague or peer relationship	Negative dynamics in the teams causing stress.	Supportive team relationships were important for managing stress at work
Line manager relationship	Line managers relationships were not perceived as supportive for some participants and contributed to stress	Line managers were reported as creating a better supportive climate for work wellbeing for others
**Theme: management practices**		
Autonomy		Most felt able and motivated to make constructive suggestions related to how they run their tasks
Recognition and career advancement	Lack of praise and recognition from management as a source of stress	Opportunities for career development were cited as a resource
Communication	Lack of platform to discuss and air out grievances	
**Theme: meaningfulness of work**		
Making a difference in clients’ lives		Most were passionate about, and felt rewarded by, differences they make in their clients’ lives.
Support service delivery		Non patient facing participants discussed how they feel their services enhance other’s jobs which motivated them
Theme: family/personal issues	Stresses relating to relationships, including family, negatively affected workplace wellbeing.	

### Working conditions

Participants referred to working conditions as a source of stress.

Workload- Participants described feeling overwhelmed by the high demands of work, pressure to meet targets, and the lack of time to meet those demands. Participants were concerned that they were not able to provide quality services.

*When the line is too long, I feel overwhelmed, because the patients also start complaining that they have been here for this long. Female linkage officer.*

There were, however, some participants who described their workload in a positive way, pointing out that the amount of work was generally reasonable.

Work-Life Balance – Furthermore participants described the heavy workload to compromise private life; some participants mentioned lack of leisure time and strained family relationships.

Difficulties taking leave due to staff shortages contributed to this.

*If I take 3 weeks my work will suffer and my work will wait for me and when I come back, it would have piled up and make me suffer work burnout because of the pressure. Female Decanting Officer.*

Emotional effect of interacting with patients - Participants described feeling very confident in their work and capable of effectively dealing with problems, however some reported being vulnerable or emotionally affected by patients’ situations. One participant shared their experiences:

*When I see someone who is struggling it makes me feel sad and I keep thinking about that person and it makes me stressed. I cannot even sleep at home because I keep thinking about that person. Female Retention Counselor.*

Some participants reported that they fail to separate themselves easily from emotions and difficult cases especially those involving children and sexual abuse.

Mental Health Support- Most participants felt there is a lack of consideration for employees’ mental wellbeing. Some participants were not aware of systems for debriefing or support, while others felt that support was inadequate. All participants reported needing additional debriefing and mental health support. Many participants bemoaned that they do not have a platform to share their burdens with colleagues.

*There is nothing much to be done about it because they do not even offer us debriefing….. So, I continue as if nothing has happened – Female Enrolled nurse.*

Nature of funding - Participants reported that they were constantly concerned about losing their jobs and were concerned about losing income due to funding uncertainties.

Regarding salary, many participants reported that the remuneration received for their work is not commensurate with the effort they put into their roles or skill set possessed. There was a general appreciation of having an income, but regardless of this, they felt that the workload did not match their salary.

*My stress level is aggravated by the salary. My skill level does not match my salary. Male Data Capturer*.

### Work relationships

This theme relates to relationships that participants have with colleagues within their organization, partner organizations and with their line managers, and the influence that such relationships can have on work wellbeing.

Relationship with colleagues within their organization – All participants described a great experience of support from their team and were positive about their relationships with their peers. This relationship appeared to be one of the key sources of support and encouragement for many interviewed. Most participants valued teamwork with their colleagues, finding it motivating. Participants also described informal peer support, discussing cases, and supporting and guiding each other.

*I think that [relationship with other team members] is what keeps me going. I think with that I must applaud them. Even when I am feeling down or feel like quitting, they always support you. Female Enrolled Nurse.*

Relationship with line manager: There was a mix of positive and negative relationships with managers. Some participants described their managers as supportive in both work-related and personal factors:

*My manager is cool, he sits down with me and if there is a problem, he calls me and tries to find out what is wrong, and we try and find a solution to that problem. Male Monitoring and Evaluation officer.*

Those who felt negatively toward their line manager described them as unavailable:

*Sometimes you just have burnout, and we need to speak to her, but they do not allow us. Female Linkage officer.*

Relationship with Colleagues from host DOH- The relationship with the host was reported as difficult by most respondents, with only a few considering them to be positive. Respondents reported being undervalued and unfairly treated by colleagues from the host DOH. One particpant mentioned:

*They sideline us and sometimes they will have the meetings and do not include us, and in those meetings, they will change things without including us and that is not okay because we work together. Female enrolled nurse.*

### Management practices

The management practices theme relates to autonomy, communication, recognition and career advancement.

Autonomy- Another source of wellbeing was the perception of autonomy. Most felt able and motivated to present constructive suggestions related to how they run their tasks to their line managers. Participants indicated that they found their work satisfying as they are involved and free to express their opinion and have control when it came to managing their work.

*The good thing about where I am right now is that I am given the opportunity to think out of the box and be independent to try and to find ways to interpret the data. It is because I get to liaise with different people, and I am trusted a lot with my work. Male Monitoring and Evaluation Officer.*

Communication- some participants stated that management lacks an open-door policy that would allow for consultation and staff involvement. Participants shared a desire for a platform where they can discuss critical issues and receive communication regarding changes in the organization.

*There will not be any communication that addresses you as an individual but instead hear it from the grapevine or passages- Female Linkage officer.*

Recognition and career advancement - Participants’ discussions on this theme were varied with both praise and criticism of their organization. Participants described promotions as well as career development through gaining on-the-job experience as motivating. While others reported feeling unappreciated by management, expressed a lack of support and understanding and shared that management did not give them sufficient recognition, implying a sense that some roles were unimportant and not respected as part of the team.

### Meaningfulness of work

The rewarding nature of their roles was an important source of job satisfaction among participants. All participants described this as an important aspect of their job. Participants indicated that they found purpose and meaning in their work through making a positive difference in the lives of patients. One participant shared:

*You know that young people are scared to come to the clinic so I work with young people who can relate, and I am a bridge to the clinic…. It makes me happy because it means that I can change someone’s life. Female Youth Ambassador.*

This theme applied to all roles, and those employees who are not patient facing also found meaning in their work. Data related and supervisory role participants accounted for meaningful work in terms of offering services for the betterment of other providers in their workspace.

*The impact that I have on the counselors, the support that I provide to them, I see the changes in their work, if I was not there for them, they will be lost. Male HIV Testing Services Coordinator*.

### Personal factors

Participants acknowledged exposure to social stresses relating to relationships and family. Such experiences affect participants’ work wellbeing negatively and can affect the colleagues they work with. Conversely, when participants were asked about mechanisms for managing stress, family and social support were commonly mentioned as important factors.

*I face situations, especially with kids’ cases, and I talk to my husband at home, and I become well after those conversations. Female Linkage officer.*

Other mechanisms mentioned by participants included prayer, meditation, exercise, breaks, alcohol, and counseling. Some simply accepted the stressful situation and endured the work with no stress-reduction strategy.

#### Participants suggested interventions

Participants were asked to suggest interventions for building resilience or coping with stress or burnout. Their suggestions included:

Peer-based HCW support or debriefing sessions through group meetings or sessions- Participants highlighted that when held on a regular basis, these sessions offer HCWs mental support.

Acknowledgments and financial incentives were also suggested as important factors for uplifting HCWs morale and thus supporting their wellbeing.

Other participants suggested more involvement by management to better support team work especially with external partners.

## Discussion

Our findings reveal that the prevalence of burnout syndrome among HCW in this setting is low, and we identified job resources that buffer against workplace stressors, mitigating risk of burnout. Although we report low rates of overall burnout, 40% of HCWs reported moderate/high levels of emotional exhaustion. Emotional exhaustion is the first stage leading to burnout (41), so this is a warning sign that burnout could follow. Our study highlights a need for the organization to address key drivers of burnout and foster the identified resources to promote HCW wellbeing and prevent vulnerable staff from progressing to a state of burnout. Burnout that would impact their own mental health and their ability to provide quality care to service users.

The low levels of overall burnout from our survey are inconsistent with previous studies conducted on different groups of HCWs (physicians, nurses, community health workers, midwives, and pharmacists) in LMIC using the MBI-HSS scale, which reported a prevalence ranging from 11.3 to 86.2% (25). Our findings are closer to those of studies from Ethiopia (42) and China (43) conducted among mixed primary health care workers with 3.3 –3.8% levels of burnout. The difference in findings could be attributed to the inclusion of non-patient facing HCWs in our study. Previous studies have cautioned that response profiles on the MBI-HSS are dependent on occupational contexts with regard to the nature of client-service work conducted by respondents (44). The scores in each MBI-HSS dimension [moderate to high emotional exhaustion (42.78%), high to moderate depersonalization (15.98%), and low to moderate personal accomplishment (39.69%)] are comparable to rates observed in frontline PHC service delivery providers including physicians, nurses, pharmacists, and community health workers in various outpatient health care settings including HIV care clinics in a number of LMICs (5). These studies, which include South African studies, found similar rates of moderate to high emotional exhaustion (range 27.4–99.6%), moderate to high depersonalization (13.3–98.0%), and low to moderate personal accomplishment (20.3–47.9%).

We found that roving positions were more likely to experience high emotional exhaustion and low professional accomplishment similar to a previous study on travel nurses (45). Job positions that require traveling or roving could contribute to burnout due to work-life imbalance (45) and stress (47). According to (48), consistently working side by side with familiar teammates builds a sense of belonging that may protect wellbeing, and lack of team identification is emotionally demanding and distressing (48). Roving positions may not foster team identification as easily as fixed positions.

Our qualitative findings suggest that job rewards and demands in our setting have a collective positive balance on most participants. Participants had high job demands characterized by high workload. The health sector in general is understaffed and lacks skills, creating a high workload (49). Because of the present economy, many organizations are finding themselves trying to accomplish more with fewer employees, subsequently affecting workload, employee–employer relationships, motivation, and job security (50). Furthermore, participants revealed that there was inadequate support for mental health promotion, consistent with findings from studies conducted in similar settings (51).

Relational issues with host department of health staff were reported as emotionally demanding and distressing. The effect of social undermining within healthcare teams (52), along with communication problems, absence of trust and unclear roles, increases the risk of burnout (53). NPO-government collaboration is a complicated process as it involves multiple organizations, each with their own priorities and characteristics (54). It is important that leadership teams of all stakeholders acknowledge this and actively support their frontline staff to be effectively incorporated into facility-based teams with greater efforts to build positive relationships across organizations.

While there were still many challenges to overcome, the study found participants to have high job resources. The majority of the respondents found great meaning in their roles, consistent with other studies of HCWs in primary care settings (51, 55) and linked to their job satisfaction. People working in data teams were most likely to have low professional accomplishment scores, suggesting they may need additional support in this area. Similar to previous literature, our participants indicated autonomy, support of supervisors, and positive relationships with colleagues to improve their job satisfaction (56). Moreover, the participants reported adopting strategies to bolster wellbeing including meditation, counseling, and getting support from friends and family. These job resources and strategies act together to promote HCW wellbeing at work, even in settings with high job demands. Our findings are similar to other studies, (40, 57) that have highlighted that there are factors (resources) which can act as buffers to stress at work and may reduce the effect of demands placed on the individual.

Finally, an objective of this study was to make recommendations to guide interventions to improve wellbeing and decrease burnout. Teamwork should be promoted through encouraging communication, promoting interaction (for example, through team meetings), providing opportunities for social activities and peer support groups, and training in good team dynamics. Acts of acknowledgment and appreciation through employee reward and recognition programs should be implemented, reflecting the respect and dignity that HCWs associate with their profession (58).

### Limitations

The survey response rate was low (20%), although in line with other survey-based studies (59), as the response rate to online surveys is often low depending on the content and length of the questionnaire, especially among health professionals (60). People who are particularly struggling at work may be less likely to respond to survey request. The use of self-report measures in the survey is prone to biases arising from participants providing socially desirable responses and cultural factors. Concerns have been raised about how the MBI-HSS operationalizes burnout and there may be different cultural interpretations of questions related to the construct of burnout (8). However, the mixed method approach made it possible for in-depth analysis to complement survey findings. For example, despite the survey revealing low rates of burnout syndrome, the qualitative approach revealed the existence of high job demands, such as work overload, challenges with management, difficult workplace relationships, and the need for workplace mental health support.

Another limitation of this study is the absence of a comprehensive psychometric evaluation conducted to support the findings of the MBI-HSS. While the study focused on assessing burnout levels, the lack of accompanying psychometric analyses may undermine the robustness of our findings. This limitation should be considered when interpreting the study’s findings and implications. We recommend that further investigations are undertaken to establish the validity of the burnout measures employed in this setting.

## Conclusion

We found low rates of burnout syndrome, but fairly high rates of emotional exhaustion, and lack of personal accomplishment, as well as working conditions that negatively impacted wellbeing. Participants were exposed to high job demands, such as work overload, challenges with management, and difficult workplace relationships. However, most participants found value in their health care roles and the difference they made in people’s lives. This meaning and teamwork within the organization promoted resilience and coping skills to protect against burnout. This NPO and other health sector employers should strengthen workplace interventions that promote resilience and help to prevent burnout, including by fostering these job resources, recongnizing, and, where possible, seeking to minimize job demands. Structured employee interventions should be designed to build and maintain resilience in a challenging environment.^1^

## Data availability statement

The raw data supporting the conclusions of this article will be made available by the authors, without undue reservation.

## Ethics statement

The studies involving humans were approved by Faculty of Health Sciences Research Ethics Committee at the University of Witwatersrand (WITS HREC Medical: M210417) and Provincial Health Research Committee (NHRD REF. NO.: GP_202110_050). The studies were conducted in accordance with the local legislation and institutional requirements. The participants provided their written informed consent to participate in this study.

## Author contributions

KR, NM-Z, and MB developed the protocol. AJ and FB conducted the qualitative data collection. NS and NM-Z led the quantitative and qualitative data analysis, respectively, with input from AJ, FB, KR, MB, and ND. NM-Z wrote the first draft of the manuscript. AJ, FB, KR, and ND reviewed and revised the draft manuscript. All authors reviewed and approved the final manuscript as submitted.

## Footnotes

^1^A distress protocol was followed for participants should they become distressed and confidential counseling services were provided free of charge from the Employee Assistance Program (EAP).

## Glossary of titles

**Table:**

Group	Job title	Description
Clinicians	Enrolled Nurse	Provide support to health facilities with the implementation of HIV Testing Service, Tuberculosis screening, promotion of family planning and provision of sexual reproductive health services, enhanced adherence counseling and facilitation of adherence clubs and other Central Chronic Medicines Dispensing and Distribution (CCMDD) modalities
	Professional Nurse	Support initiation of HIV infected clients on ART, PrEP, STI treatment and ensure full implementation of the 95 95 95 activities.
Counselors	Linkage Officer	Responsible for HIV Testing Services and ensure that proper linkage to care of all HIV infected patients is done according to DoH guidelines
	Retention Officer	Responsible to enhance retention in care with specific focus on patients (re)initiating ART, patients that are virally unsuppressed despite effective ART and patients at high risk of disengaging from care
Data Team	Monitoring and Evaluation (M&E) Officer	Lead and coordinate the implementation of M&E systems focused on data collection, analysis, and reporting of results within Anova APACE supported facilities
	Data Capturer	Carry out data-related admin work, capture patient’s files, generate defaulter reports and compile monthly statistics
Decanting	Decanting	Implementing repeat prescription collection strategies (RPCS) – adherence clubs, spaced fast lane appointments and decentralized medicine options for patients on chronic treatment, targeting those that are stable
Other	Youth Ambassador	Promote healthy behaviors and prevent risk behaviors in adolescents through; the implementation of targeted interventions at individual, facility, and community level to enhance uptake of HIV-testing and linkage to and retention to treatment and uptake of prevention services such as PrEP and sexual reproductive health and services.
	HIV Testing Services Coordinator	Provide leadership and oversight to Anova Counselors and HIV Testing Services Testers allocated to work at primary health care facilities, hospital, and community
	Clinical Technical Advisor	Provide technical and operational support regarding diagnosis, clinical management and treatment of HIV and TB programs

## Abbreviations

DoH: Department of Health
HCW: health care worker
LMIC: low and middle-income country
MBI: Maslach Burnout Inventory
MBI-HSS: Maslach Burnout Inventory Human Services Survey
NPO: Non-Profit Organization
PEPFAR: President’s Emergency Plan for AIDS Relief
PHC: Primary Health Care
PLHIV: People Living with HIV
SSA: Sub-Saharan Africa
USAID: United States Agency for International Development
WHO: World Health Organization
