# Supplementary material for: Workplace wellbeing among health care workers providing HIV services in primary care in Johannesburg: a mixed methods study
Source: Front Public Health. 2023 Oct 20;11:1220301. doi: 10.3389/fpubh.2023.1220301 (PMC10643173; doi:10.3389/fpubh.2023.1220301)
Supplement: Supplementary file 1 [file Data_Sheet_1.docx]

Maslach Burnout HSS Survey questionnaire

**MASLACH BURNOUT SURVEY QUESTIONNAIRE**

**Study Title:** ***Measuring Burnout among Anova Health Care Workers in Johannesburg during COVID-19.***

**NB:** Please cross (x) the most appropriate response to indicate your answer.

**DATE** _________________________________

**DEMOGRAPHICS**

1. Age

1. Sex

- Female
- Male
- Transgender
- Other
- Prefer not to say

1. Job Title

1. Facility Name

5. Place of work. Mark where you have spent the most time working. Pick only one.

- PHC
- CHC
- Hospital
- Office
- Work Remotely (Home)
- Community Work

6. How often do you interact with patients?

- Never
- Occasionally
- Once a month
- Once a week
- A few times a week
- Everyday

7. In what way do you interact with patients?

- Consult
- HIV Testing
- Handing over files/cards
- I do not interact with clients directly
- Other:

**Maslach Burnout Inventory Questionnaire (patients used for HSS adaptation)**

| **Emotional Exhaustion** | Never  (0) | A few times a year  (1) | Monthly  (2) | A few times a month  (3) | Every week  (4) | A few times a week  (5) | Every  Day  (6) |
| --- | --- | --- | --- | --- | --- | --- | --- |
| 1. I feel emotionally drained at work |  |  |  |  |  |  |  |
| 2. I feel used up at the end of the workday |  |  |  |  |  |  |  |
| 3. I feel fatigued when I get up in the morning and have to face another day on the job |  |  |  |  |  |  |  |
| 4. Working with people all day is really a strain for me |  |  |  |  |  |  |  |
| 5. I feel burned out from my work  6. I feel frustrated by my job  7. I feel I’m working too hard on my job  8. Working with people directly puts too much stress on me  9. I feel like I’m at the end of my rope |  |  |  |  |  |  |  |
|  |  |  |  |  |  |  |  |
|  |  |  |  |  |  |  |  |
|  |  |  |  |  |  |  |  |
|  |  |  |  |  |  |  |  |
| **Personal Accomplishment** |  | | | | | | |
| 10. I can easily understand how my patients feel about things |  |  |  |  |  |  |  |
| 11. I deal very effectively with the problems of my patients |  |  |  |  |  |  |  |
| 12. I feel I’m positively influencing other people’s lives through my work |  |  |  |  |  |  |  |
| 13. I feel very energetic |  |  |  |  |  |  |  |
| 14. I can easily create a relaxed atmosphere with my patients  15. I feel exhilarated after working closely with my patients  16. I have accomplished many worthwhile things in this job  17. In my work, I deal with emotional problems very calmly |  |  |  |  |  |  |  |
|  |  |  |  |  |  |  |  |
|  |  |  |  |  |  |  |  |
|  |  |  |  |  |  |  |  |
| **Depersonalization** |  | | | | | | |
| 19. I feel I treat some patients as if they were impersonal ‘objects’ |  |  |  |  |  |  |  |
| 20. I’ve become more callous toward people since I took this job |  |  |  |  |  |  |  |
| 21. I worry that this job is hardening me emotionally |  |  |  |  |  |  |  |
| 22. I don’t really care what happens to some patients  23. I feel patients blame me for some of their problems |  |  |  |  |  |  |  |
|  |  |  |  |  |  |  |  |
|  |  |  |  |  |  |  |  |
|  | Never  (0) | A few times a year  (1) | Monthly  (2) | A few times a month  (3) | Every week  (4) | A few times a week  (5) | Every  Day  (6) |
| **Emotional Items- Involvement** |  | | | | | | |
| 24. I feel similar to my patients in many ways |  |  |  |  |  |  |  |
| 25. I feel personally involved with my patients’ problems |  |  |  |  |  |  |  |
| 26. I feel uncomfortable about the way I have treated some patients |  |  |  |  |  |  |  |
|  |  |  |  |  |  |  |  |

Thank you for your participation. If you would like to participate in a follow-up interview regarding burnout, please feel free to provide your contact details below and a member of the research team will follow-up with you to schedule the interview telephonically or in-person.

Would you like to participate in the follow-up interview?

- Yes, I would like to participate in the interview

Name:

Contact Number:

Email:

- No, I do not want to participate in the interview

**END**

**Scoring & Interpretation Key – MBI-HSS**

**Instructions.** For each column, transfer your response (0-6) from the original measure to the three columns below. Only transfer numbers to the unshaded/ungreyed spaces. Then, sum each column and place that number in the space provide below. This number represents your score for that dimension. Guidelines for interpretation can be found on the right side of the sheet.

*Emotional Exhaustion* *Depersonalization* *Professional Accomplishment*

|  | **How Often** | | |  |  | **How Often** | | |  |  | **How Often** | |  |
| --- | --- | --- | --- | --- | --- | --- | --- | --- | --- | --- | --- | --- | --- |
|  |  | **0-6** | |  |  |  | **0-6** | |  |  |  | **0-6** |  |
| 1. | | _________ | |  |  | 1. |  | _________ |  |  | 1. | _________ |  |
| 2. | | _________ | |  |  | 2. |  | _________ |  |  | 2. | _________ |  |
| 3. | | _________ | |  |  | 3. |  | _________ |  |  | 3. | _________ |  |
|  |  |  |  |  |  |  |  |  |  |  |  |  |  |
|  | 4. |  | _________ |  |  | 4. |  | _________ |  |  | 4. | _________ |  |
|  | 5. |  | _________ |  |  |  |  |  |  |  |  |  |  |
|  |  |  |  |  |  | 5. | _________ | |  |  |  |  |  |
|  |  |  |  |  |  |  |  |  |  |  | 5. | _________ |  |
|  |  |  |  |  |  |  |  | |  |  |  |  |  |
|  | 6. | _________ | |  |  | 6. |  | _________ |  |  | 6. | _________ |  |
|  |  |  | |  |  |  |  |  |  |  |  |  |  |
|  | 7. |  | _________ |  |  | 7. |  | _________ |  |  | 7. | _________ |  |
|  |  |  |  |  |  |  |  |  |  |  |  |  |  |
|  | 8. | _________ | |  |  | 8. |  | _________ |  |  |  |  |  |
|  |  |  |  |  |  |  |  |  |  |  | 8. | _________ |  |
|  |  |  | |  |  |  |  |  |  |  |  |  |  |
|  | 9. |  | _________ |  |  | 9. |  | _________ |  |  | 9. | _________ |  |
|  | 10. |  | _________ |  |  |  |  |  |  |  |  |  |  |
|  |  |  |  |  |  | 10. | _________ | |  |  | 10. | _________ |  |
|  | 11. |  | _________ |  | 11. | | _________ | |  |  | 11. | _________ |  |
|  |  |  |  |  |  |  |  | |  |  |  |  |  |
|  | 12. |  | _________ |  |  | 12. |  | _________ |  |  | 12. | _________ |  |
|  |  |  |  |  |  |  |  |  |  |  |  |  |  |
|  | 13. | _________ | |  |  |  |  |  |  |  |  |  |  |
|  |  |  |  |  |  | 13. |  | _________ |  |  | 13. | _________ |  |
| 14. | | _________ | |  |  |  |  |  |  |  | 14. | _________ |  |
|  |  |  |  |  |  | 14. |  | _________ |  |  |  |  |  |
|  |  |  | |  |  |  |  |  |  |  |  |  |  |
|  | 15. |  | _________ |  |  | 15. | _________ | |  |  | 15. | _________ |  |
|  |  |  |  |  |  |  |  | |  |  |  |  |  |
|  | 16. | _________ | |  |  | 16. |  | _________ |  |  | 16. | _________ |  |
|  |  |  | |  |  |  |  |  |  |  |  |  |  |
|  | 17. |  | _________ |  |  | 17. |  | _________ |  |  | 17. | _________ |  |
|  | 18. |  | _________ |  |  | 18. |  | _________ |  | 18. | | _________ |  |
|  | 19. |  | _________ |  |  | 19. |  | _________ |  | 19. | | _________ |  |
|  |  |  |  |  |  |  |  |  |  |  |  |  |  |
|  | 20. | _________ | |  |  |  |  |  |  |  |  |  |  |
|  |  |  |  |  |  | 20. |  | _________ |  |  | 20. | _________ |  |
|  |  |  | |  |  |  |  |  |  |  |  |  |  |
|  | 21. |  | _________ |  |  | 21. |  | _________ |  |  | 21. | _________ |  |
|  | 22. |  | _________ |  |  |  |  |  |  |  |  |  |  |
|  |  |  |  |  |  | 22. | _________ | |  |  | 22. | _________ |  |
|  |  |  |  |  |  |  |  |  |  |  |  |  |  |

EE Sum______ Dep Sum______ PA Sum______

Categorization:


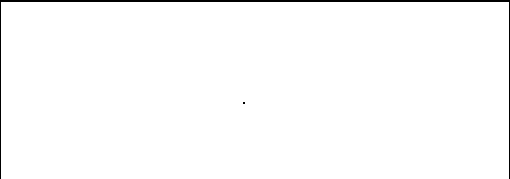


Emotional Exhaustion, Human Services &

Educators Forms

|  | Frequency |
| --- | --- |
| High | 27 or over |
| Moderate | 17-26 |
| Low | 0-16 |

Categorization:


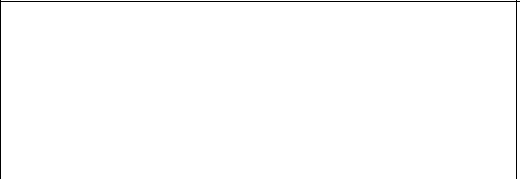


Depersonalization, Human Services Form

|  |  | Frequency |
| --- | --- | --- |
| High |  | 13 or over |
| Moderate |  | 7-12 |
| Low |  | 0-6 |
|  |  |  |
|  | Categorization: | |
|  | Personal Accomplishment,* | |
|  | Human Services Form | |
|  |  | Frequency |
| High |  | 39 or over |
| Moderate |  | 32-38 |
| Low |  | 0-31 |

**MBI-General Survey**: Copyright ©1996, 2016 Wilmar B. Schaufeli, Michael P. Leiter, Christina Maslach & Susan E. Jackson.

**MBI-Human Services Survey**: Copyright ©1981 Christina Maslach & Susan E. Jackson.

**MBI-Educators Survey**: Copyright ©1986 Christina Maslach, Susan E. Jackson & Richard L. Schwab.

All rights reserved in all media. Published by Mind Garden, Inc., www.mindgarden.com

Qualitative Interview Guide

Thank you so much for taking time to participate in this discussion on burnout. Burnout is potentially very serious for workers, their clients, and the larger institutions in which they interact. Burnout is composed of 3 main pillars: Emotional exhaustion is the most recognizable – the sense of overwhelming fatigue or having nothing left to give at the end of a shift; Depersonalization often expressed by cynicism, detachment and loss of empathy for your patients/clients; and finally a low sense of personal accomplishment – the feeling you aren’t making an impact at your work or “why bother…nothing is going to change”.

As you are aware, we conducted a survey recently, and the results from that survey showed that burnout is an important issue among Anova health workers at in Johannesburg. Moving forward we would like to implement strategies that may bring about positive change and help prevent or reduce burnout. To this end, we are conducting in-depth discussions with selected health care workers to understand more about the issues within our work environment and your day to day work experiences.

1. Please tell me about yourself
   1. What is your current occupation?
   2. How long have you been working with Anova?
   3. What is your highest qualification?
   4. What is your gender?
   5. What is your marital status?

In the introduction I provided a brief overview of burnout. We are now going to discuss burnout among health workers, and how this has changed if any amid COVID-19? Our discussion will dwell on risk factors of burnout; workload, supportive work community, control, fairness and recognition.

1. Please tell me about your job?
   1. Please describe your work routine
   2. Do you feel you job is right match for your skills?
   3. Do you feel that your role is important?
      1. Do you think you make a difference in the day to day operations of your job? Why or why not?
      2. Do you think you make a difference to the community/ your clients? Why or why not?
   4. Do you feel your workload is manageable?
   5. Are you satisfied with the salary for your profession? If no, how do you feel about it?
2. Now I want us to discuss your job during COVID-19.
   1. Has your routine job changed since the beginning of the coronavirus outbreak? How?
   2. What is the impact of the Covid-19 virus on your workload?
   3. How do you feel working during COVID-19? *Probe: are you afraid of contracting the virus, are you afraid on infecting your family, are you concerned over PPE shortages*.
   4. What is your view on how Covid-19 virus response is being managed at Anova and clinic? Do you feel that there is clear leadership?
3. Let’s talk about resources because resources are essential to doing your job. Tell me about the level of resources you have available in your job.
   1. Staff
   2. Equipment and supplies
   3. Training
   4. What happens when you don’t have enough resources—how does management/ colleagues respond?
   5. Has the availability of resources changed since the beginning of the coronavirus outbreak? How?
4. Team dynamics can have an impact on developing and perhaps preventing burnout. Thinking about your work environment can you describe your relationship with coworkers and how important those relationships are?
   1. *Probe: Do you feel a group cohesiveness or do people just do what they want?*

*What impact does this have on you? How does this make you feel?*

1. Let us talk about freedom to participate in implementation of interventions. How involved are you in decision-making? Does your input into decision-making or lack of affect you? Why or why not?
2. Do you feel you have autonomy (chance to try own methods of doing a job related task) in your job? Why or why not? Is workplace autonomy important to you?
3. Do you think clients value the services that you render? Please explain? How does this make you feel?
   1. What about colleagues, do you think they value services that you render? Please explain? How does this make you feel?
   2. What about management, do you think they value services that you render? Please explain? How does this make you feel?
4. We are now going to talk about emotional impact of your work.
   1. Are you emotionally affected by problems of clients? Please explain your answer- describe a stressful/difficult/complicated situation which you think can be related to burnout?
   2. Are you emotionally affected by problems of workplace/organisation? Please explain your answer- describe a stressful/difficult/complicated situation which you think can be related to burnout?
   3. If you are affected how do you cope?
   4. Do you think burnout has been intensified amid COVID-19? Please explain.

- 1. Are there measures in place at work to help you decrease your emotional impact? What are those measures?

1. We are now going to talk about your personal/family life.
   1. Does stress at work affect your personal life? If so, can you tell me about these.
   2. Do you have enough time to relax/ attend to family responsibilities?
   3. Are there any support systems outside your workplace to help you decrease your emotional impact? What are those support systems?
2. We have talked today about challenges in your work environment. We are now moving our attention to thinking about possible strategies that can help reduce or prevent burnout.
   1. What intervention may be useful in preventing burnout? How could it work in the current workplace?

Before concluding, is there anything else you’d like to discuss or think we should consider?

Thank you for your time.
